# The use of depot antipsychotics in patients with dual diagnosis of psychosis and substance use disorder is associated with improved quality of life, better general clinical outcome and fewer hospitalizations

**DOI:** 10.1192/j.eurpsy.2025.893

**Published:** 2025-08-26

**Authors:** E. I. Koiliari, I. Mouzas, G. I. Detorakis, E. L. Pasparakis

**Affiliations:** 1 Department of Psychiatry, General Hospital of Agios Nikolaos of Lasithi, Agios Nikolaos of Lasithi; 2 Department of Pathology, Laboratory of Alcohology of the Medical School of the University of Crete; 3 Department of Pathology, University General Hospital of Irakleion (PAGNI), Herakleion, Greece

## Abstract

**Introduction:**

Co-occurring substance use disorders (SUDs) among individuals with schizophrenia are a prevalent and complex psychiatric comorbidity, which is associated with increased symptom severity, worsened illness trajectory and high rates of treatment non-adherence. Recent evidence suggests that the use of depot antipsychotics may provide an effective treatment option for individuals with this dual-diagnosis.

**Objectives:**

Hypothesis testing: “Depot antipsychotics are associated with i) reduced hospitalizations, ii) improved quality of life and iii) improved patient functionality in dual diagnosis of psychosis and SUD”.

**Methods:**

68 patients in community of Eastern Crete (Greece) participated (Male to Female ratio corresponds to 2.4:1). All of them manifested psychosis (24 with F.20 ICD-10 and 44 with F29.0 ICD-10). The median age was 41 years. 29.41% had dual diagnosis of psychosis and alcohol use disorders, 7.35% had dual diagnosis of psychosis and cocaine use disorders, while 26.47% had dual diagnosis of psychosis and cannabis Use disorder. 80.88% were on aripiprazole LAI, 8.82% on paliperidone LAI, 2.94 % on risperidone LAI and 7.35% on haloperidol LAI. For the evaluation of our hypotheses the instruments WHOQOL-BREF questionnaire and the CGI-S scale were used. The quality of life and the functionality of the patients and also the number of their hospitalizations were compared in each patient, before the initiation of the LAI medication and during the active treatment period. The minimum of follow-up period was 6 months.

**Results:**

In our sample of 68 patients with depot antipsychotics therapy administrated at least for 6 months: a) Hospitalizations decreased statistically significantly from 1.01 ±1.54 to 0.01±0.12 (Paired Samples Wilcoxon Signed Rank Test p-value<<0.001), b) The CGI-S score decreased statistically significantly from 5.72 ±0.88 to 2.94±1.33 (Paired Samples Wilcoxon Signed Rank Test p-value<0.001), c) The score of the WHOQOL-BREF scale increased statistically significantly from 0.57 ±0.53, to 3.35±0.84 (Paired Samples Wilcoxon Signed Rank Test p-value<0.001). The same sample with depot antipsychotic treatment administrated at least for 3 months: a) The CGI-S score decreased statistically significantly from 5.72 ±0.88 to 2.34±0.89 (Paired Samples Wilcoxon Signed Rank Test p-value<0.001), b) The score of the WHOQOL-BREF scale increased statistically significantly from 0.57 ±0.53, to 2.3±0.89 (Paired Samples Wilcoxon Signed Rank Test p-value<0.001).

**Conclusions:**

Depot antipsychotics medication significantly reduces the number of hospitalizations, when patients with psychosis and substance use disorders remain in therapy at least for 6 months. In addition, the administration of depot therapy, at least for 6 months, improves the quality of life and the functionality of these patients.

**Disclosure of Interest:**

None Declared

